# Feasibility and Outcomes of Perirectal Spacer Implantation in Previously Treated Prostate Cancer Patients Undergoing Salvage Proton Therapy Radiation

**DOI:** 10.1016/j.adro.2025.101985

**Published:** 2026-02-09

**Authors:** Irini Yacoub, Keyur Mehta, Daniel Gorovets, Shaakir Hasan, J. Isabelle Choi, Arpit M. Chhabra, Stanislav Lazarev, Madhur Garg, Charles B. Simone

**Affiliations:** a New York Proton Center, New York, New York; b Department of Radiation Oncology, Memorial Sloan Kettering Cancer Center, New York, New York; c Department of Radiation Oncology, Montefiore Einstein Cancer Center, Bronx, New York; d Department of Radiation Oncology, Ohio Health, Columbus, Ohio; e Department of Radiation Oncology, Mount Sinai Medical Center, New York, New York

## Abstract

**Purpose:**

Treatment of recurrent prostate cancer following prior therapy is challenging due to the risk of toxicities to previously irradiated normal tissues, particularly the rectum. Proton therapy offers a conformal approach that may reduce this risk. Perirectal spacers have been shown to further minimize rectal dose in primary treatment, but the ability to be placed and their value in the retreatment setting have not been thoroughly evaluated to date. The purpose of this study was to evaluate feasibility of rectal spacer placement after prior pelvic radiation or other local treatments for prostate cancer.

**Methods and Materials:**

We conducted a retrospective review of 30 consecutive patients with biopsy-confirmed, locally recurrent prostate adenocarcinoma treated with salvage proton therapy at the New York Proton Center (2020-2024). All patients had prior local therapies including high intensity focused ultrasound (HIFU), brachytherapy (BT), external beam radiation therapy (EBRT) or combination BT and EBRT, and underwent perirectal spacer placement (SpaceOAR or Barrigel). Spacer feasibility, maximum and minimum separation distances, rectal symmetry, rectal Dmax (% of prescription dose), and gastrointestinal toxicities were assessed. Toxicity was graded using Common Terminology Criteria for Adverse Events, version 5.0.

**Results:**

Median patient age was 74 years, and the median time from prior therapy was 102 months. Spacer placement was successfully completed in 28 of 30 (93%) patients. Two placements were aborted due to excessive fibrosis. The median maximum and minimum midaxial anterior-posterior separations were 10.8 mm and 3.5 mm, respectively. Median rectal Dmax was 91.55% (range, 44%-102.4%). Seven patients had Dmax >100%, including 3 with prior HIFU. At a median follow-up of 12.5 months, gastrointestinal toxicities were limited to grade 1 diarrhea (n=3) No grade ≥ 2 toxicities or procedural complications were reported.

**Conclusions:**

This is the first reported study evaluating the feasibility of perirectal spacer implantation in men with prior treatment to the pelvis, undergoing salvage radiation with protons. Our report shows perirectal spacer implantation is feasible and safe in patients undergoing salvage proton therapy, including those with prior EBRT or BT. Although technically more challenging in HIFU-treated patients, successful placement was achieved in the majority, with minimal toxicity and favorable dosimetric outcomes, although longer follow-up is needed.

## Introduction

Several local treatment options exist for patients with localized prostate cancer, including prostatectomy, radiation therapy (RT) with or without androgen deprivation therapy, and focal ablative treatments. Despite advances in these treatment modalities, biochemical progression rates range from 15% to 57%.^1^, ^2^, ^3^ In men who have received prior RT, treatment in the salvage setting is limited by risk of further toxicity to surrounding previously irradiated nontarget tissue but include external beam radiation, brachytherapy, and surgery, with or without androgen deprivation. Often in the reirradiation setting proton beam radiation is offered as the external beam option. Proton beam radiation offers a highly conformal treatment modality to the prostate through modulation of the Bragg peak, allowing for sparing of surrounding normal tissue, which is especially critical in the reirradiation setting.^4^ Additionally, the use of protons allows for safe delivery of definitive doses in patients whose disease has recurred and would otherwise render itself to palliative management.^5^ Verma et al^6^ evaluated clinical outcomes and toxicities of proton RT for reirradiation and demonstrated favorable survival outcomes with acceptable toxicities.

Since their introduction, gel spacers are increasingly used in men undergoing RT to the prostate. Growing evidence has demonstrated their general safety and ability to reduce rectal dose exposure and resulting toxicity.^7^^,^^8^ However, in men who have received prior local therapies to the prostate, there is often dense fibrosis between Denonvillier’s fascia and the mesorectal fascia, typically making the dissection of posterior planes extremely complex.^9^ The insertion of perirectal spacers in men who have been previously irradiated or received local ablation, therefore, can be difficult and has not been thoroughly evaluated. However, the use of spacers in this patient population may be even more beneficial to reduce the risk of serious toxicities resulting from multiple courses of irradiation to the pelvis. This is the first reported study evaluating the feasibility of perirectal spacer implantation in men who have received prior treatment to the pelvis or prostate undergoing salvage radiation with proton therapy.

## Methods and Materials

We retrospectively reviewed the charts of consecutive patients receiving salvage proton radiation at the New York Proton Center for recurrent prostate cancer. Patients included in this study had biopsy-proven locally recurrent prostate adenocarcinoma in the prostate gland without midline posterior extra-capsular extension. Patients underwent placement of either SpaceOAR Hydrogel (Polytethylene Glycol (PEG)-based hydrogel) or Barrigel (hyaluronic acid-based) per physician preference.

Preoperatively, patients received prophylactic antibiotics for coverage of standard skin organisms. Patients also received Fleet rectal enemas to optimize ultrasound visualization of the fascial plane. Prior to insertion, patients received either monitored anesthesia care or local anesthesia using 1% lidocaine, according to their preference. During the insertion, a transrectal ultrasound probe was placed with the patient in the dorsal lithotomy position, and a biplanar view (axial and sagittal) of the prostate was confirmed. For the SpaceOAR Hydrogel placement, visualization of the fascial space was confirmed on ultrasound, and then an 18 gauge needle was passed transperineally between the rectum and prostate. The needle position was evaluated on both sagittal and axial views to confirm midline placement. The space was then hydrodissected using a small amount of saline (typically up to 10 cm^3^) to confirm the spacer would be deployed in the proper plane. Then, the hydrogel system was injected into the space. When the Barrigel spacer was used, a transperineal needle and syringe with the gel was deployed directly into the space without need for prior hydrodissection. If the insertion was deemed feasible, most patients received 6 to 9 ml of Barrigel. The amount was at the discretion of the performing radiation oncologist if they felt the spacing was sufficient per patient anatomy.

Postprocedural treatment planning used both a computed tomography scan and magnetic resonance imaging (MRI), if there were no MRI contraindications. T2 weighted MRI was used to evaluate perirectal spacer placement symmetry using a semiqualitative method previously described by Fischer-Valuck et al.^10^ Reirradiation dose was determined at the discretion of the treating physician. The primary endpoint of this study was gel separation width between prostate and rectum (measured on MRI in the midaxial plane for maximum and minimum separation width, as well as at the level of the midgland). Secondary endpoints included rectal Dmax, spacer symmetry, and acute and late gastrointestinal (GI) toxicity. Rectal Dmax was collected as a percentage of the prescription dose. Toxicity was assessed per Common Terminology Criteria for Adverse Events version 5.0 garnered from electronic medical record documentation including weekly on-treatment visit, 3-month follow-up, and 6-month follow-up notes.

## Results

Table 1 describes patient and treatment characteristics. Between July 2020 and December 2024, 30 locally recurrent prostate cancer patients who were previously treated with external beam RT (EBRT) alone (n = 22), brachytherapy alone (n = 4), combined EBRT + brachytherapy (n = 1), or high-intensity focused ultrasound (HIFU) (n = 3) were treated with salvage intensity modulated proton beam RT. The most prescribed regimen was proton stereotactic body RT delivered to 3500 cGy in 5 fractions and 7020 cGy in 26 fractions (Table 1). Mean and median American Urologic Association urinary symptom scores prior to salvage radiation were 8.97 and 8.5, respectively (range, 0-23). Seventy-three percent of patients had positron emission tomography prostate-specific membrane antigen imaging prior to salvage radiation.

**Table 1:** Patient and treatment characteristics

Patient characteristic	No. (%)
Age (median, range, y)	74 (56-84)
Prior therapy	
EBRT alone	22 (73%)
HIFU	3 (10%)
BT alone	4 (13%)
EBRT + BT	1 (3%)
Dose Re-RT	
3000 Gy RBE	2 (7%)
3500 Gy RBE	20 (67%)
3625 Gy RBE	2 (7%)
5625 Gy RBE	1 (3%)
6000 Gy RBE	1 (3%)
7020 Gy RBE	4 (13%)
Spacer placed	
Barrigel	19 (63%)
SpaceOAR	9 (30%)
Aborted	2 (7%)
Prostate size (median, range, mL)	30 (14-103)
PSMA prior to salvage RT	22 (73%)
AUA urinary symptom scores prior to salvage radiation (median, range)	8.5 (0-23)
SHIM scores prior to salvage radiation (median, range)	5.5 (1-26)

*Abbreviations:* AUA = American Urologic Association; BT = brachytherapy; EBRT = external beam radiation therapy; HIFU = high intensity focused ultrasound; PSMA = prostate-specific membrane antigen; RBE = relative biological effectiveness; RT = radiation therapy; SHIM = sexual health inventory for men.

**Figure 1:**
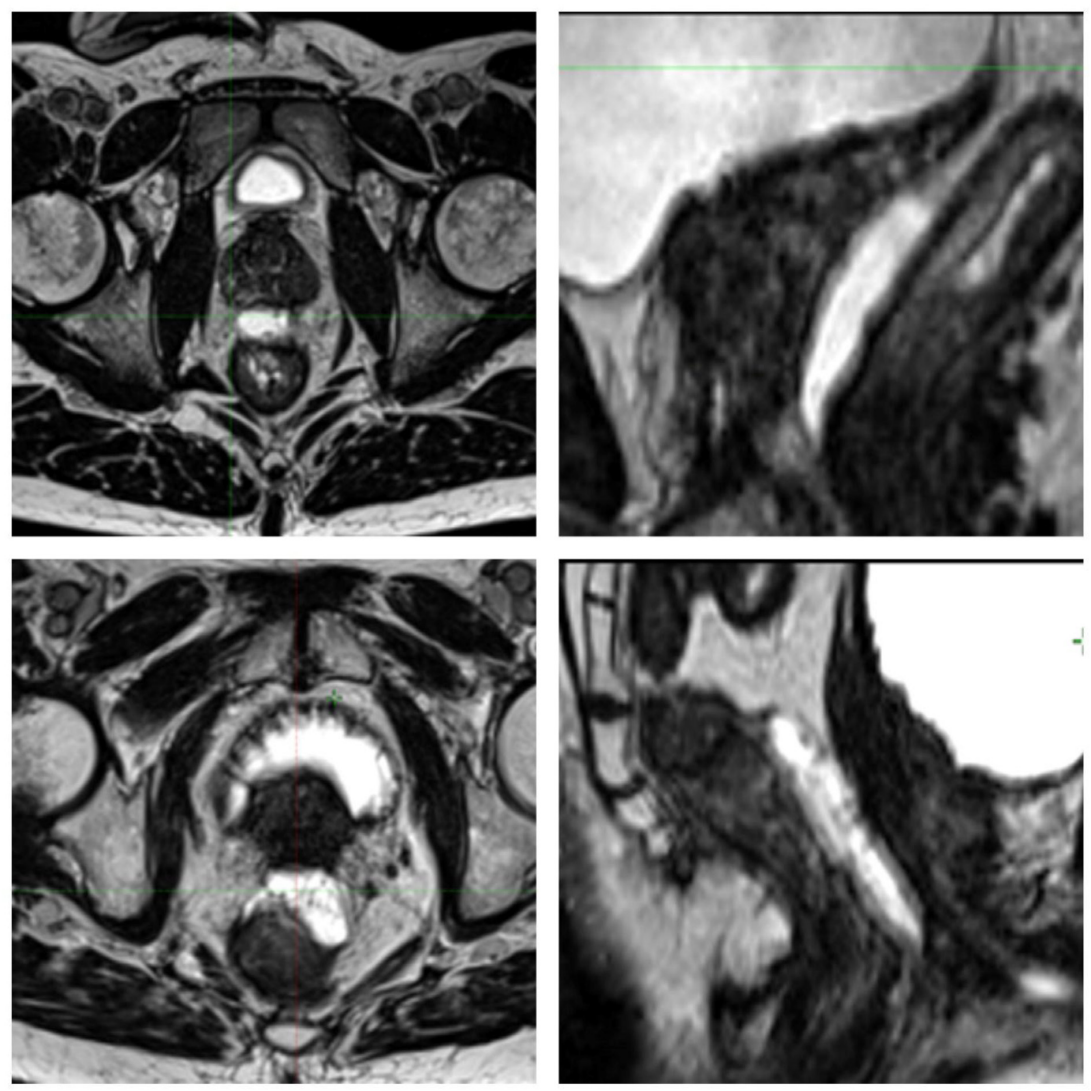
Spacer placement examples. Axial (left) and sagittal (right) examples of Barrigel spacer (top) and SpaceOAR (bottom) placement.

The median patient age at the time of retreatment was 74 years (range, 56-86), and patients had a median interval from initial treatment of 102 months (range, 18-592) and median prostate size of 30 cm^3^ (range, 14-103). Of the 30 attempted spacer insertions, 2 (7%) were aborted due to excessive tissue resistance (1 in a patient with prior HIFU, and 1 in a patient with prior EBRT). The 2 other patients previously receiving HIFU were notably more difficult for spacer implantation than the remaining patients receiving prior EBRT and/or brachytherapy. SpaceOAR was used in 9 cases, and Barrigel was used in the remaining 19 cases (figure 1). There were no postprocedural complications within 30 days of the procedure.

The median maximum separation in the midaxial anterior-posterior direction was 10.8 mm (range, 5.5-19.3), median minimal separation was 3.5 mm (range, 0.5-6.4), and median midgland separation was 8 mm (range, 2-12). The average, median, and range of spacer volume placed for all feasible patients was 6.7 cc, 6.05 cc, and 2-13.8 cc, respectively. Mean, median, and range spacer volume was 5.77 cc, 10.1 cc, and 2-13.8 cc, respectively, for SpaceOAR patients, and 9.4 cc, 5.1 cc, and 2.9-13.3 cc for Barrigel patients. PEG hydrogel was found in all but 3 axial MRI slices (3/84, 4%) while scoring hydrogel symmetry. Overall, using the previously described method by Fischer-Valuck et al., a score of 1 and 2 were seen in 21 of 28 (75%) of superior slices, 25 of 28 (89%) of midgland slices, and 26 of 28 (93%) of inferior slices. Regarding overall symmetry per patient, 5 of 28 (17.9%) had SYM 1, 3 of 28 (11%) had SYM 2, 7 of 28 (25%) had SYM 3, 13 of 28 (46%) had SYM 4, and no patients had SYM 5.

The median rectal Dmax, as described in the methods section, was 91.55% (range, 43.9%-102.4%), and median mean rectal dose was 11.2% (range, 1.0%-26.7%). Seven patients had Dmax >100%, 3 of whom had prior HIFU, including the patient previously treated with HIFU who had an aborted spacer procedure. Excluding the aborted case, the remaining 5 patients with rectal Dmax > 100% had a median minimal separation of 4.5 mm (range, 2-9), and a median maximum separation of 11 mm (range, 5.5-14). Median minimal separation for patients with rectal Dmax < 100% was 4.75 mm (range, 2-6.5) and median maximal separation of 10.5 mm (range, 6.1-16.6).

Three patients developed acute grade 1 diarrhea. Neither of the patients with the 2 aborted spacers had any acute or chronic bowel toxicities. At a median follow-up of 12.5 months (range, 2-51), there were no grade 2 to 5 acute or late toxicities in any of the patients.

## Discussion

There are currently no well-established guidelines for the treatment of locally recurrent prostate cancer after RT or other local ablative treatments. Nonsurgical management using brachytherapy,^11^ HIFU,^12^ and EBRT^13^ are all reasonable options in the retreatment setting.

In the reirradiation setting, it is often difficult to meet normal tissue, namely rectal, dose constraints, often necessitating a reduction in target coverage of a potentially radioresistant recurrent tumor. Maximal therapeutic benefit of reirradiation can be achieved using perirectal spacers to allow sparing of this organ. The first randomized trial by Mariados et al^7^ evaluated the dosimetric and clinical benefits of hydrogel spacer in men undergoing image guided intensity modulated RT. The authors reported a 99% placement success rate, with an average space of 12.6 mm in the spacer group. There was a significant reduction in late rectal toxicity severity in the spacer group, and no late rectal toxicity greater than grade 1 in the spacer group. In that study, patients who received prior RT were excluded, precluding evaluation of placement feasibility in this cohort of patients. A similar randomized study using hyaluronic acid-based spacers demonstrated similar outcomes with rectal spacing, including improved rectal dosimetry and reduced acute grade 2 or higher GI toxicity.^8^ In that trial, spacer placement was rated difficult or very difficult for 7% of patients. Similar to the SpaceOAR trial, that study excluded patients who had prior local prostate cancer treatment, including cryotherapy and EBRT. As such, the feasibility of placement of hyaluronic acid-based spacers in the previously treated pelvis is unclear.

The use of proton beam radiation allows for normal tissue sparing through modulation of the Bragg peak, leading to conformal prescription dose delivery to the target volume while minimizing excess dose to normal tissues. This normal tissue sparing property of proton therapy can be particularly beneficial in the reirradiation setting to potentially reduce the risk of late toxicities of retreatment. In our feasibility study of retreatment patients, we demonstrated a 93% successful implantation rate, with a median maximal separation in successful placements of 10.8 mm. Despite the known possibility of dense fibrosis between Denonvillier’s fascia and the perirectal fascia in patients with previous local therapy, a minimal separation of at least 2 mm was achieved in this study. Given the conformality provided by proton beams, even a space of 2 mm between the prostate and the rectum may facilitate a dose reduction to the rectum. In patients with successful spacing, the highest rectal Dmax seen was 101.9%, which is within tolerance for rectal constraints. Spacer symmetry was also evaluated using the method previously described by Fischer-Valuck et al^10^ in de-novo prostate patients. We used the same method of assessment in patients with prior prostate treatment or pelvic radiation and we demonstrated that up to 93% of our patients had gel at midline and within 1 cm of midline. Although midline-to-lateral symmetry is less critical for rectal protection than achieving sufficient anterior-posterior separation to elevate the prostate away from the rectum, our study demonstrates that such symmetry remains achievable in this salvage population.

In conclusion, this is the first known study to report on the feasibility of perirectal spacer placement in patients with prior local therapies for prostate cancer. We demonstrated a 93% successful implantation rate, using both PEG-based and hyaluronic acid-based spacers. In patients who received prior local therapies and thus may have fibrotic tissue in the periprostatic space, the use of gel spacing appears feasible, without concerns for high rates of procedural complications, and it is associated with satisfactory rectal Dmax and minimal GI toxicity. Larger studies and longer follow-up are needed to validate these findings.

## Disclosures

None.
